# Supplementary material for: miR-183/96/182 Cluster Regulates the Development of Bovine Myoblasts through Targeting FoxO1
Source: Animals (Basel). 2022 Oct 17;12(20):2799. doi: 10.3390/ani12202799 (PMC9597811; doi:10.3390/ani12202799)
Supplement: Supplementary file 1 [file animals-12-02799-s001.zip › animals-1862736-supplementary.pdf]

Supplementary Table S1： Primer Information in our work

pCK-FoxO1-wt-F	CCGCTCGAGGAGTGAGCAAGCGAGCAAGCAG
pCK- FoxO1-wt-R	ATAAGAATGCGGCCGCGTGTAGTGAGTTTGGCACTTC
pCK- FoxO1-mut-F	CCGCTCGAGGAGTGAGCAAGCGAGCAAGCAG
pCK- FoxO1-mut-R	ATAAGAATGCGGCCGCTTTGGCTGTTCATTGTAATGAATTTCCAAACGCAC
bta-miR-183-RT	GTCGTATCCAGTGCAGGGTCCGAGGTATTCGCACTGGATACGACCAGTGAA
bta-miR-96-RT	GTCGTATCCAGTGCAGGGTCCGAGGTATTCGCACTGGATACGACAGCAAAA
bta-miR-182-RT	GTCGTATCCAGTGCAGGGTCCGAGGTATTCGCACTGGATACGACAGTGTGA
bta-miR-183/96/182-R	GTGCAGGGTCCGAGGT
bta-miR-183-F	GGCGTATGGCACTGGTAGAA
bta-miR-96-F	GGCGTTTGGCACTAGCACAT
bta-miR-182-F	GGCGTTTGGCAATGGTAGAAC
GAPDH-F	CACTGAGGACCAGGTTGTCT
GAPDH-R	TGTCGTACCAGGAAATGAGC
β-actin-F	GTCATCACCATCGGCAATGAG
β-actin-R	AATGCCGCAGGATTCCATG
U6-F	GCTTCGGCAGCACATATACTAAAAT
U6-R	CGCTTCACGAATTTGCGTGTCAT
PCNA-F	AACCTCACCAGCATGTCCAA
PCNA-R	CCAACGTGTCCGCGTTATCT
CDK2-F	TCTTTGCTGAGATGGTGACCC
CDK2-R	CATCTTCATCCAGGGGAGGC
Cyclin D1-F	ATGAAGGAGACCATCCCCCT
Cyclin D1-R	CGCCAGGTTCCACTTGAGTT
P21-F	AGGGCACGTCTCAGGAGGA
P21-R	CAGTCTGCGTTTGGAGTGGTAG
MYOD-F	AACACTACAGCGGCGACTC
MYOD-R	GCTGTAGTCCATCATGCCGT
MYOG-F	CCAGTACATAGAGCGCCTGC
MYOG-R	AGATGATCCCCTGGGTTGGG
MYF5-F	TCTATCTCTCTGCTGTCCAGGC
MYF5-R	GTACTCAGAGGGCGAGAACTG
FOXO1-F	CAGATTTACGAGTGGATG
FOXO1-R	CAGATTATGACGAATTGAAT
MYOD-F	AACACTACAGCGGCGACTC
MYOD-R	GCTGTAGTCCATCATGCCGT
MYOG-F	CCAGTACATAGAGCGCCTGC
MYOG-R	AGATGATCCCCTGGGTTGGG
MYHC-F	TGCTCATCTCACCAAGTTCC
MYHC-R	CACTCTTCACTCTCATGGACC
MYF5-F	TCTATCTCTCTGCTGTCCAGGC
MYF5-R	GTACTCAGAGGGCGAGAACTG
